# Spectral Investigations of Fluorescence Tracers in Automotive and Aviation Fuels under Cryogenic Conditions

**DOI:** 10.3390/s24030724

**Published:** 2024-01-23

**Authors:** Matthias Koegl, Jonas Vogler, Lars Zigan

**Affiliations:** 1 Institut für Thermodynamik, Professur für Energiewandlung, Fakultät für Luft-und Raumfahrttechnik, Universität der Bundeswehr München (UniBw M), D-85577 Neubiberg, Germany; jonas.vogler@unibw.de (J.V.); lars.zigan@unibw.de (L.Z.); 2 Erlangen Graduate School in Advanced Optical Technologies (SAOT), Friedrich-Alexander-Universität Erlangen-Nürnberg (FAU), D-91054 Erlangen, Germany

**Keywords:** two-color LIF technique, liquid temperature, Nile red, Eosin-Y, cryogenic conditions, fuels

## Abstract

This study investigated spectral laser-induced fluorescence signals of dyes in fuels for automotive and aerospace applications under low temperatures and cryogenic conditions down to 183 K. For this purpose, a fluorescence chamber was developed based on cooling with liquid nitrogen. The design enabled a minimal inner chamber temperature of 153 K. Furthermore, the applicability of two-color LIF for liquid thermometry was evaluated under these conditions. The temperature determination was based on the temperature-sensitive fluorescence intensity ratio of the special dyes doped into the fuels determined in suitable spectral regions, which represented common bandpass filters. For this purpose, the fluorescence signals of the dye doped into the gasoline and jet fuel surrogate isooctane were tested as well as blends of isooctane and the ethanol biofuels E20 (comprising 80 vol.% isooctane and 20 vol.% ethanol), E40, and E100. Additionally, a realistic multi-component fuel Jet A-1 mixed with a suitable fluorescence dye was investigated. E100 was doped with Eosin-Y, and the remaining fuels were doped with Nile red. Temperature-dependent spectral LIF intensities were recorded in the range of 183 K–293 K, which simulate extreme environments for aerospace and automotive applications. Frozen fuel–dye mixtures cause significant extinction effects and prevent sufficient signal detection at low and cryogenic temperatures, defining the detection limit. A temperature decrease led to a spectral shift in the emission peaks of E100 doped with Eosin-Y toward shorter wavelengths, while the spectra of mixtures doped with Nile red were shifted toward longer wavelengths. The suggested bandpass filters produced the temperature-sensitive intensity ratio (the average over the temperature interval) of the dyes with the largest sensitivity for Jet A-1 (5.2%/K), followed by E100 (4.95%/K), E40 (4.07%/K), E20 (3.23%/K), and isooctane (3.07%/K), even at cryogenic temperatures.

## 1. Introduction

Combustion engines, aircraft turbines, and especially rockets may be operated under extreme environmental conditions. Consequently, the liquid fuel may be injected at very low or even cryogenic temperatures, which is especially true for rocket conditions. Cold starts can be very challenging and critical operating conditions for any type of combustion engine. For example, in Antarctica, ambient temperatures can easily drop down to 180 K–200 K. This may be below the freezing point of the fuel, or the viscosity and surface tension are too high for the proper injection and atomization of the fuel. This could result in fuel film formation on the walls or very large liquid fuel structures or droplets and worsened mixture formation, fuel consumption, as well as pollutant emissions. Consequently, detailed knowledge of the injection and fuel atomization processes in engines under cold-start conditions under these extreme conditions is beneficial for the development and optimization of such combustors, which is especially true for combustion-engine-driven military and emergency vehicles and systems (generators, etc.).

In general, the reliability, efficiency, and pollutant formation are governed by the whole combustion process chain, including liquid fuel atomization, evaporation, mixing, and reaction. This process chain is not fully understood, and the introduction of multi-component biofuels leads to even higher complexity as the atomization process may be significantly altered. Consequently, knowledge of local droplet sizes, droplet temperatures, and information about liquid films formed on the combustor walls is relevant. The presence of large droplets and/or droplet films on engine components (e.g., gasoline engines) leads to delayed evaporation, inhomogeneous mixture formation, and diffusive combustion, which causes increased pollutant formation and emissions (such as soot, carbon monoxide, CO, and nitric oxides, NO_x_) and should be prevented. Consequently, fuel film and droplet temperature information can be used for an optimized combustor design and atomization strategy under cold-start conditions to achieve the best possible efficiency and low pollutant emissions.

Numerous in situ techniques for spray characterization have been developed over the last decades including Mie scattering, phase Doppler interferometry (PDI), shadowgraphy, and laser-induced fluorescence (LIF) [1,2,3,4]. Techniques based on LIF are the focus of the present paper and, therefore, are described in more detail. LIF may enable a planar determination of droplet size and temperature [5]. LIF/Mie droplet sizing (often termed “*d*_32_ droplet sizing”) is used to determine the planar droplet size distribution (the Sauter mean diameter (SMD, *d*_32_) within a spray [6,7]. Furthermore, multiple non-invasive techniques for the characterization of the liquid film thickness have been developed in the last decades. Very thin films (0.1–3 µm) can be measured with refractive-index-matched (RIM) imaging [8,9]. Laser-induced fluorescence is mainly used for thicker films (10 µm–100 µm) [10,11,12,13,14,15,16], laser absorption spectroscopy for film thicknesses of up to 1600 µm [17,18,19], and laser absorption techniques, such as laser light absorption, for film thicknesses of up to 5 mm [20]. The two-color LIF technique may enable the simultaneous measurement of the film temperature and film thickness. It is also widely applied for droplet or spray thermometry. The ratio-based two-color LIF technique for thermometry utilizes the temperature-dependent shift and broadening of the LIF spectrum. The intensity ratio of two well-selected spectral detection channels (realized using individual bandpass filters in front of two cameras (see Section 2.1)) changes with temperature and enables the determination of the planar temperature distribution after adequate calibration. With a known temperature, the film thickness can be derived with the intensity distribution of one of the two detection channels after calibration.

A fluorescence signal can be created in two ways: using a so-called “tracer” dissolved in a liquid [21] or using the liquid itself (e.g., the aromatic components in lubricants or fuels) [22,23,24]. The fluorescence signal is governed by the spectral properties (absorption and emission) of the tracer and many more properties (such as the solvent, temperature, utilized illumination source (e.g., a laser, LED, and the respective excitation wavelength), bandwidth, and irradiation). The temperature sensitivity is mainly governed by the tracer itself and the polarity of the solvent. Laser-induced phosphorescence (LIP) could also be applicable for liquid and two-phase thermometry, but the seeding of particles into the liquid is very challenging, and particle agglomeration may occur, which may lead to large measurement errors [23,25].

Widely used LIF dyes for temperature measurements in liquids include Rhodamine B, Eosin-Y, fluorescein, pyrromethene, coumarin, and Nile red [5,26,27,28,29]. Coumarin dissolved in ethanol shows a high temperature sensitivity but must be excited in the UV wavelength range, where certain fuel components (e.g., toluene, gasoline, etc.) show large absorption cross-sections as well [27]. Rhodamine B works well in combination with ethanol and water [30,31,32,33,34]. Fluorescein can either be dissolved in water or ethanol [35,36,37]. Pyrromethene is mainly used in alkanes (e.g., dodecane), ketones (such as 3-pentanone), and alcohols [32,38,39,40,41,42]. Nile red is used as a dye for non-polar fuels or oils like isooctane, kerosene, Fragoltherm F12, and Marlotherm LH. Nile red enables the determination of the mixture composition of, e.g., butanol/decane and ethanol/isooctane mixtures [43,44].

Until now, only a few investigations in the field of combustion have dealt with the detection of film thickness [8,14] and temperature [5] based on laser-induced fluorescence, however, not simultaneously and not under cryogenic conditions. For example, Park et al. [45] measured the film temperature and thickness separately in the piston crown of a direct-injection spark-ignition engine. The authors used a fiber-based LIF method, where a single fiber was used for excitation and signal collection (photo-multiplier-based). They excited the tracer N,N′-Bis(2,5-di-tert-butylphenyl)-3,4,9,10-perylenedicarboximide (BTBP) dissolved in isooctane at 488 nm and utilized the signal intensity ratio at 515 nm and 532 nm (realized with bandpass filters) for the film temperature measurements (358 K–407 K). The film thickness (<10 µm) was measured with 2,3-hexanedione dissolved in isooctane excited at 457.9 nm, since both components showed similar evaporation behavior.

The present work focuses on the capability of measuring the fluorescence of tracers in liquid automotive and aviation fuels under low- and cryogenic-temperature conditions. A special emphasis was put on the development of a two-color LIF thermometry technique under extreme-temperature conditions. The goal was to use a single fluorescent dye in combination with two spectral detection channels, which is capable of determining the liquid temperature of fuel films and sprays. For this purpose, a spectral investigation of isooctane, E20 (80 vol.% isooctane and 20 vol.% ethanol), E40, E100, and Jet A-1 doped with either Eosin-Y or Nile red in terms of absorption and emission measurements was carried out. First, the influence of the dye concentration on the absorption and emission spectra was determined. Second, an optically accessible cell was designed and manufactured for fluorescence studies under low-temperature conditions down to 183 K. The cell and liquid fuels were cooled with an adjustable liquid nitrogen flow. Third, temperature-dependent spectral emission measurements were carried out, and suitable filters were selected and validated. The temperature sensitivities were calculated for the investigated mixtures. Finally, integral signals at very low temperatures were determined to evaluate the photo-physical effects and the applicability of the technique for imaging under those very challenging conditions.

## 2. Materials and Method

### 2.1. Measurement Principle

A brief overview of the LIF principles is given in the subsequent paragraph. Further information can be found in [46,47,48]. The fluorescence signal *I*_LIF_ of a tracer molecule is described with the following working equation [49]:

(1) $$I_{\mathrm{LIF}}∼\frac{E_{\mathrm{Pulse}}}{A\cdot h\nu }\cdot n_{\mathrm{Tracer}}\cdot V\cdot \sigma_{\mathrm{abs}}\left(\lambda ,T\right)\cdot \phi_{\mathrm{LIF}}\left(\lambda ,T,p,\chi_{i}\right)\cdot \eta_{\mathrm{opt}}\cdot \frac{\Omega }{4\pi }$$

The equation can be separated into excitation (due to the absorption of photons), the emission of fluorescence photons, and detection. *E*_Pulse_/(*A*⋅*hv*) describes the number of photons with respect to the laser light sheet. *E*_Pulse_ is defined as the laser pulse energy. *n*_Tracer_ is defined as the number density of the tracer molecules within the measurement volume. *σ*_abs_ is the absorption cross-section, which is excitation wavelength (*λ*)- and temperature (*T*)-dependent and defines the probability of photon absorption per tracer molecule. The fluorescence quantum efficiency *Φ* is dependent on the excitation wavelength (*λ*), the temperature (*T*), the pressure (*p*), and the composition of the ambient gas *χ_i_*. Since the fluorescence is equally distributed within the volume, the detection angle *Ω* has to be taken into account. Furthermore, the fluorescence intensity is dependent on the optical efficiency *η*_opt_ of the detection system.

The temperature in the present work was determined using a two-color detection scheme. This approach utilizes the temperature-dependent change in the emission spectra. An increase in temperature leads to a spectral shift and broadening. This behavior can be particularly seen in aromatic molecules. The signal of a laser-excited fluorescence tracer is simultaneously detected with two spectral channels, CH1 and CH2. Appropriate bandpass filters separate the detected spectral bands and are selected in a way to ensure the maximum sensitivity (change in the intensity ratio with temperature in %/K) and sufficient signal intensity. The intensity ratio *r* of these two detection channels based on Equation (1) can be described as follows:

(2) $$\begin{array}{l} r=\frac{I_{CH1}}{I_{CH2}}=\frac{{\left[\frac{E_{\mathrm{Pulse}}}{A\cdot h\nu }\cdot n_{Tr}\cdot V\cdot \sigma_{\mathrm{abs}}\left(\lambda ,T\right)\cdot \phi_{\mathrm{LIF}}\left(\lambda ,T,p,\chi_{i}\right)\cdot \eta_{\mathrm{opt}}\cdot \frac{\Omega }{4\pi }\right]}_{CH1}}{{\left[\frac{E_{\mathrm{Pulse}}}{A\cdot h\nu }\cdot n_{Tr}\cdot V\cdot \sigma_{\mathrm{abs}}\left(\lambda ,T\right)\cdot \phi_{\mathrm{LIF}}\left(\lambda ,T,p,\chi_{i}\right)\cdot \eta_{\mathrm{opt}}\cdot \frac{\Omega }{4\pi }\right]}_{CH2}}\\ =\frac{{\left[\phi_{\mathrm{LIF}}\left(\lambda ,T,p,\chi_{i}\right)\right]}_{CH1}}{{\left[\phi_{\mathrm{LIF}}\left(\lambda ,T,p,\chi_{i}\right)\right]}_{CH2}} \end{array}$$

In the signal ratio, the laser illumination, the tracer number density, the measurement volume, and the absorption cross-section can be reduced. The influence of the detection efficiency and the detection angle cancel out, too. Only the ratio of the fluorescence quantum efficiencies remains in the equation.

### 2.2. Fluorescence Spectroscopy Setup

**Figure 1:**
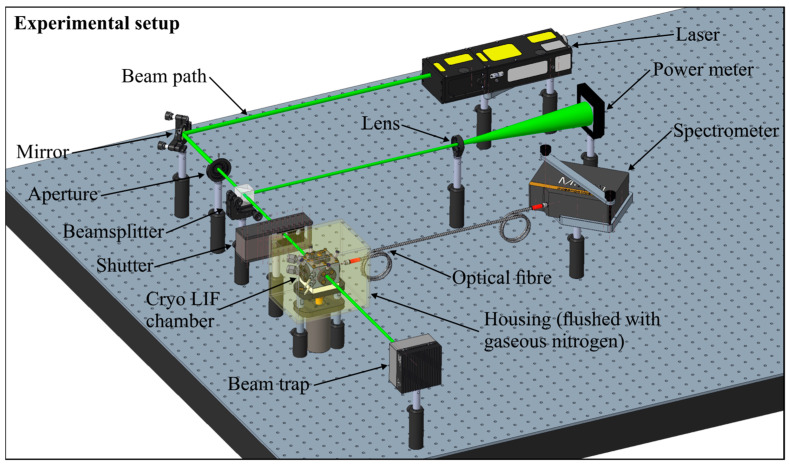
Optical setup for the fluorescence measurements.

**Figure 2:**
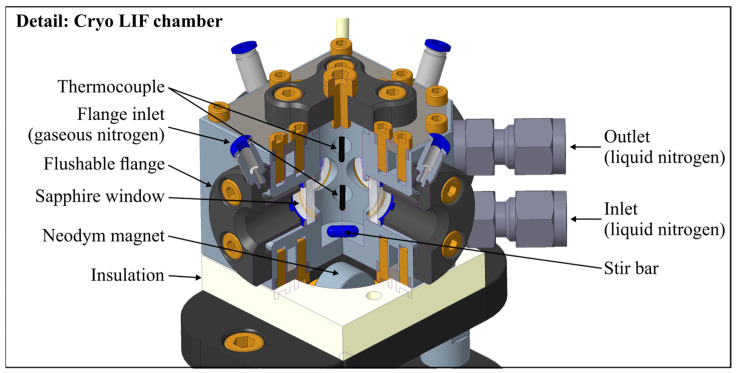
Sectioned view of the cryo LIF chamber.

The experimental setup of the fluorescence measurements is shown in Figure 1. The probe was illuminated with a pulsed Nd:YAG laser (Ultra50 model, bigSky, Mountain Village, MT, USA) with a wavelength of 532 nm, repetition rate of 10 Hz, pulse width of <8 ns, and laser fluence of 12 mJ/cm^2^. An aperture in front of the laser reduced the laser beam cross-section down to 2.8 mm. The laser beam was separated with a glass plate (transmission: 0.875; reflection: 0.125), and the laser fluence was monitored simultaneously during the measurements with a power meter (model QE50LP-S-MB-INT-D0, Gentec Electro-Optics, Quebec, QC, Canada). An electrically actuated shutter enabled continuous power measurements and probe illumination only during measurements. This kept the possible photo-dissociation effects as low as possible. Finally, the transmitted beam illuminated the probe volume. The fluorescence spectra were detected with a spectrometer (model: Maya 2000-Pro, Ocean Optics, Orlando, FL, USA), with a wavelength range of 200.5 nm–1120.4 nm, 2048 pixels, a slit size of 25 µm, and an integration time of 100 ms. A total of 50 subsequent spectra were averaged for each measurement at a detection angle of 90°. The cryo LIF chamber (see Figure 2) was used for the temperature-dependent measurements and a cuvette (model: 101-10-40, Hellma Analytics; cross-section: 10 mm × 10 mm; volume: 3.5 µL) for the concentration studies. For the cryogenic measurements, the emission signal was detected via an optical fiber.

### 2.3. Cryo LIF Chamber

Earlier absorbance and fluorescence measurements of the group were conducted in a micro-cell, which was driven with a recirculating thermostat [28,43,50]. Herein, the lower temperature limit was governed by the mass flow and the viscosity of the recirculating thermostat used. In general, these devices are available for temperatures down to ~230 K. Only special cooling devices can achieve temperatures as low as ~150 K–180 K. The cooling capacity at these low cryogenic temperatures is significantly low. Due to heat loss, a conventional recirculated micro-cell driven with a thermostat can achieve temperatures down to 253 K (a conventional recirculating unit). The use of special recirculating units will limit the temperature to ~190 K. Lower temperatures can only be realized using a new design concept. The newly developed “cryo LIF chamber” is driven with liquid nitrogen from a pressurized storage tank (0.2 MPa) and is shown in Figure 2. Good optical access is ensured by four optical accessible ports (½″ sapphire windows; optical access diameter: 9 mm; inner distance between two windows: 19.1 mm). An integrated magnetic stirrer (stir bar: 8 mm × 3 mm; 1500 rpm) and two thermocouples (type K, tc-direct GmbH, Mönchengladbach, Germany) secure a homogenous temperature distribution within the cell. The integrated cooling circuits were driven with liquid nitrogen and enabled a homogeneous temperature distribution at cryogenic temperatures of the probe volume down to ~153 K. The chamber temperature was defined by the mass flow of the liquid nitrogen and the heat transfer to the surroundings. There was no special insulation of the walls since the gaseous nitrogen flow was crucial for the heat transfer of the chamber surface. The chamber was mounted on a special insulation ceramic to ensure a minimal heat transfer rate from or to the mounting area. However, very low wall temperatures lead to the condensation of water in the ambient air, which finally leads to ice formation on the cold surfaces. To avoid ice formation on the windows and in the volume between the spectrometer and the window, specially designed window flanges were used to avoid ice-based extinction effects (see Figure 2). Gaseous nitrogen was used to flush the windows and remove the ambient air (moisture) from the illumination and measurement paths. In addition, the chamber was enclosed in a square box (see Figure 1), which was also flushed with gaseous nitrogen supplied by a gas bottle. The spectral emissions were recorded with a fiber attached to the spectrometer. The temperature of the liquid-nitrogen-driven cryo LIF chamber was limited to 173 K due to convection heating on the outer wall due to the gaseous nitrogen flushing.

### 2.4. Absorption Spectroscopy Setup

The absorption measurements were carried out in a cuvette (specified in Section 2.2) in a UV/VIS spectrometer (model V-750, JASCO, Japan). The settings and specifications are provided in Table 1.

## 3. Fuels and Tracers Used

In the present study, the emission and absorption signals of the fluorescence dyes Nile red and Eosin-Y in various non-fluorescent automotive and aviation substitute fuels under cryogenic conditions were investigated. Nile red was dissolved in isooctane, E20, E40, and Jet A-1. Eosin-Y was used as a tracer for ethanol since it has a 2.3-higher absorption cross-section at 532 nm than Nile red dissolved in ethanol [51]. Isooctane is known as a well-defined surrogate fuel for gasoline. Commercially available gasoline consists of various components. Herein, up to 35 vol.% was aromatic components (e.g., toluene, benzene, or xylene). The composition varies significantly with the production facility/production batch. The same is true for aviation fuels in general. Besides isooctane, the automotive ethanol biofuel (E100) and its mixtures E20 (80 vol.% isooctane; 20 vol.% ethanol) and E40 (60 vol.% isooctane; 40 vol.% ethanol), and the aviation fuel Jet A-1 were investigated. The properties of the investigated fuels are displayed in Table 2.

Further details regarding the applied tracers can be found in refs. [28,43] for eosin Y and ref. [21] for Nile red.

## 4. Results

The results are structured as follows. Initially, the absorption and fluorescence spectra of the fuels at various tracer concentrations excited at 532 nm are analyzed. Second, the photo-dissociation effect of the dye in the mixtures is investigated. Third, the temperature-dependent emission spectra are presented, and suitable bandpass filters are selected, which should show the high temperature sensitivity of the respective signal ratio. The integral signals are evaluated at low temperatures in order to assess the LIF imaging capability in liquid films and droplets under these challenging conditions. Finally, a short discussion is provided. All spectral measurements are shown between 380 nm and 780 nm. This wavelength region is most relevant for the spectral behavior (absorption/emission) of the presented tracer–fuel mixtures.

### 4.1. Concentration-Dependent Measurements

#### 4.1.1. Absorption

**Figure 3:**
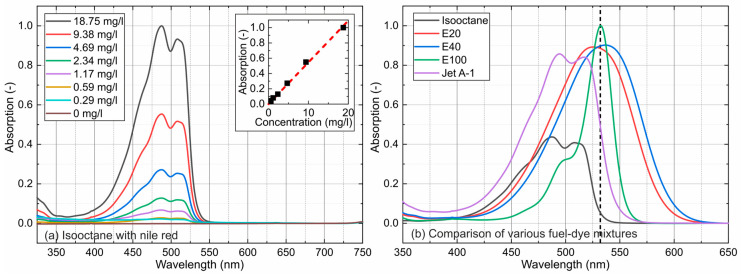
Concentration-dependent absorption spectra of Nile red dissolved in isooctane (**a**) and a comparison of various fuel–dye mixtures at a fixed dye concentration of 4.69 mg/L (**b**) and 293 K.

The absorption spectra of Nile red in isooctane (a) for various tracer concentrations and common representations of all investigated mixtures (normalized to E100 at 4.69 mg/L) (b) are displayed in Figure 3. The fuels do not absorb light in the visible wavelength range.

**Figure 4:**
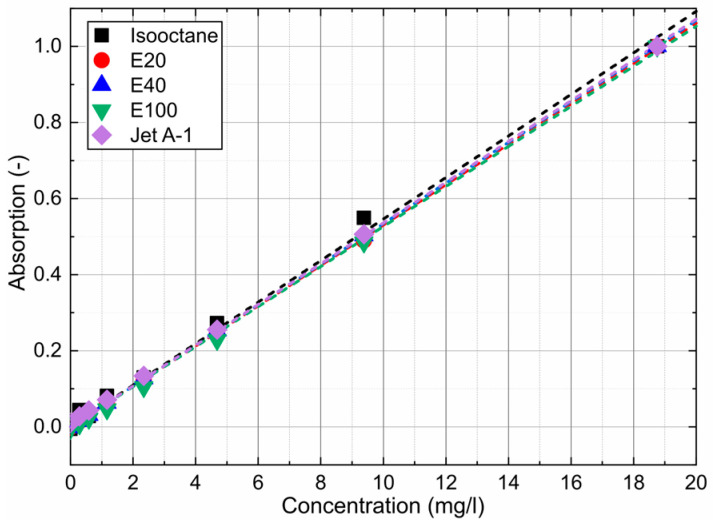
Linearity of the concentration-dependent absorption signals at a fixed dye concentration of 4.69 mg/L and 293 K.

The linearity plots of the concentration-dependent absorption signals for various solvents and the respective dyes are provided in Figure 4.

All measurements were conducted at 293 K. The absorption spectra exhibited a broad absorption between 375 nm and 650 nm and were characterized by a single peak (E20, E40, and E100) or a double peak (isooctane and Jet A-1). E20 and E40 showed a broad peak, which shifted with the increasing ethanol concentration toward higher wavelengths (E20: 525.7 nm; E40: 536.57 nm). Earlier investigations by the group showed that a small amount of an alcohol admixture (<5%) in isooctane transforms the initially double peak of isooctane into a broad single peak [44]. The previous investigations revealed an increased shift in the absorption and emission spectra with an increasing ethanol admixture, especially at low ethanol concentrations (<10 vol.%). This behavior merges the initial two characteristic spectral peaks of isooctane at very low ethanol concentrations (i.e., below 0.32 vol.%). The pronounced narrow peak of E100 (at 531.5 nm) is attributed to the different tracer Eosin-Y, which showed better absorption characteristics than Nile red at 532 nm. Nile red as well as Eosin-Y can be dissolved in ethanol. Earlier spectral investigations by the group showed that the position of the absorption maximum of Eosin-Y (~533 nm) is more appropriate (see above) than that of Nile red (548.5 nm) for an excitation wavelength of 532 nm (see, e.g., [44]). This behavior was compensated with a larger Nile red concentration in previous investigations.

Isooctane and Jet A-1 were characterized by a double peak, where the first peak was more pronounced for all investigated tracer concentrations (isooctane: peak 1—487.5 nm and peak 2—508.1 nm; Jet A-1: peak 1—494.4 nm and peak 2—516.8 nm). The comparison of the absorption spectra at a tracer concentration of 4.69 mg/L showed the highest absorption for E100 (with an absorption peak at 532.5 nm, which was very close to the laser line of the frequency-doubled Nd:YAG laser), followed by E40, E20, Jet A-1, and isooctane.

The absorption measurements in Figure 3a (inserted diagrams for isooctane) and Figure 4, which are integrated absorption signals of the individual spectra between 375 nm and 650 nm, show a linear behavior for all investigated mixtures for all concentrations. The coefficients of determination R^2^ for the linear fit curves displayed in Figure 4 are always above 0.997 and confirm the linearity of the concentration-dependent absorption measurements for all investigated dye concentrations.

#### 4.1.2. Emission

**Figure 5:**
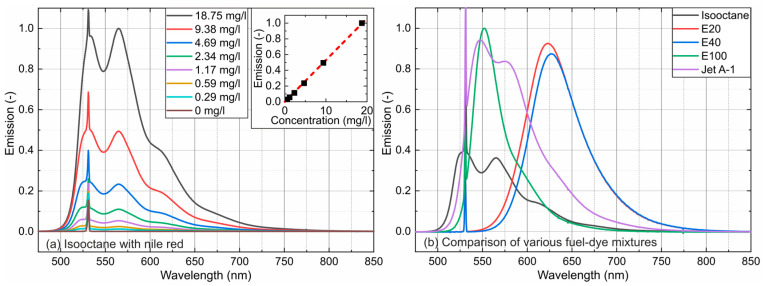
Concentration-dependent emission spectra of Nile red dissolved in isooctane (**a**) and a comparison of various fuel–dye mixtures at a fixed dye concentration of 4.69 mg/L (**b**) and 293 K.

The concentration-dependent emission measurements for various fuels mixed with Nile red (isooctane, E20, E40, and Jet A-1) and Eosin-Y (E100) up to 18.75 mg/L are provided in Figure 5.

**Figure 6:**
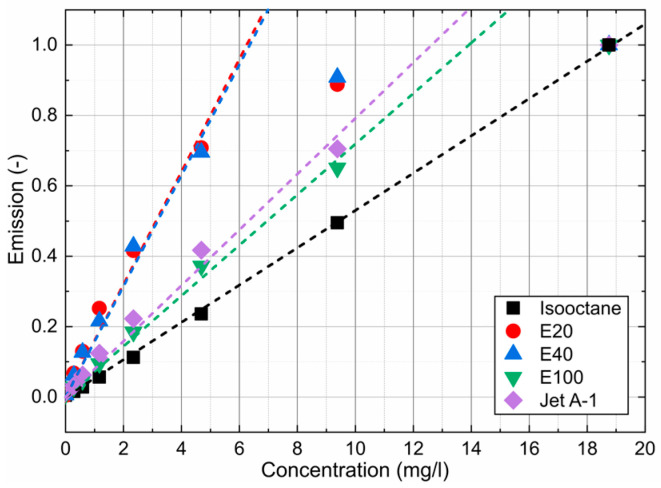
Linearity of the concentration-dependent emission signals at a fixed dye concentration of 4.69 mg/L and 293 K.

The results are normalized to the highest emission. The emission intensity of Nile red dissolved in isooctane (a) and a comparison of all investigated mixtures normalized to the highest emission at a dye concentration of 4.69 mg/L (b) are given in Figure 5, and their respective linearity is given in Figure 6. The emission peak of E100 is located at a shorter wavelength in comparison with E20 and E40, although the absorption peaks (see Figure 4) are located nearby. This is caused by the fact that E100 (Eosin-Y) and E20/E40 (Nile red) are based on different fluorescent dyes.

The dye concentration of 4.69 mg/L is the upper linearity limit of emission measurements in the cuvette due to strong absorption along the laser path and signal reabsorption (see references [24,61]). The linear function shows that all dye–fuel mixtures studied can be considered as dilute up to 4.69 mg/L.

The investigated solvents showed a broad emission between 500 nm and 800 nm and were characterized by a single peak (E20, E40, and E100) or a double peak (isooctane and Jet A-1). E20 and E40 showed broad peaks (narrower compared with the absorption), which shifted with an increasing ethanol concentration toward higher wavelengths (E20: 624.2 nm; E40: 629.0 nm). An alcohol admixture in isooctane showed the same behavior for the emission as already reported for the absorption.

Isooctane and Jet A-1 were characterized by a double peak (isooctane: peak 1—533.3 nm and peak 2—508.1 nm; Jet A-1: peak 1—552.7 nm and peak 2—578.4 nm). A comparison of the emission spectra at a tracer concentration of 4.69 mg/L (the linearity of the concentration-dependent emission for all investigated tracer–fuel mixtures (see Figure 5)) showed the largest emission peak for E100 (absorption peak at 532.5 nm (see Figure 2)), followed by Jet A-1, E20, E40, and isooctane. However, the integrated emissions were the largest for E20 and E40. Furthermore, the spectral emission was also larger for Jet A compared with isooctane. Obviously, the polar fuel components (aromatics and ethanol) increased the absorbance and, consequently, the fluorescence signal, which are directly linked.

Figure 5a (inserted diagrams for isooctane) and Figure 6 are the integrated signals of the individual spectra between 536 nm and 800 nm. Shorter wavelengths were not considered to exclude the influence of the excitation laser peak. The investigations showed a linear behavior for all investigated mixtures up to 4.69 mg/L. Only isooctane doped with Nile red showed a linear emission up to 18.75 mg/L, but at much lower signal levels compared with the other fuel–tracer mixtures. The coefficients of determination R^2^ for the linear fit curves displayed in Figure 6 are always above 0.987 and confirm the linearity of the concentration-dependent absorption measurements up to 4.69 mg/L.

The effect of photo-dissociation was also studied. This effect leads to a reduction in the LIF signal intensity with time under constant illumination and laser fluence. This effect has to be taken into account for designing test rigs in which a small volume is illuminated for a long period of time (e.g., for spectral measurements in a cuvette) or if the mixture is reused (e.g., for closed-loop cooling or spray cooling measurements) The tracer–fuel mixtures were constantly illuminated for 10 min. The fluorescence spectra were recorded every 60 s, and the spectral fluorescence intensities were summed up. Herein, only intensities between 536 nm and 800 nm were considered to avoid the influence of the excitation laser (wavelength: 532 nm). The measurements revealed that all investigated mixtures showed similar behavior with maximal signal variations of 7% in 10 min. At a laser fluence of 12 mJ/cm^2^, the effect of photo-dissociation can be neglected for short measurement durations of a few seconds for each operating point. Higher laser fluences or the usage of CW lasers or LEDs with longer illumination times would require a new investigation.

### 4.2. Temperature-Dependent Emission Spectra

**Figure 7:**
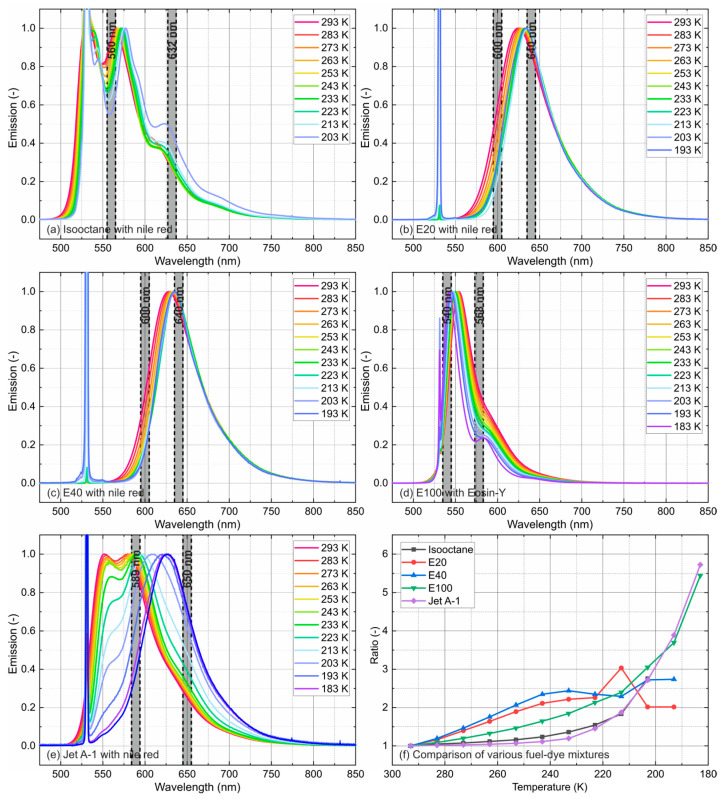
Normalized emission spectra of Nile red (4.69 mg/L) ((**a**) isooctane, (**b**) E20, (**c**) E40, (**d**) ethanol, and (**e**) Jet A-1) at various temperatures with suitable filters and corresponding temperature-sensitive intensity ratios (**f**).

The temperature-dependent fluorescence spectra with inserted bandpass filters (10 nm FWHM (full width at half maximum, which is a statistical measure used to describe the width of a normal distribution or Gaussian distribution; herein it used for the filter bandpass specification) and the corresponding temperature-dependent intensity ratios (see Equation (5)) are shown in Figure 7 and specified in Table 3. The bandpass filters were chosen in such a way that the highest possible temperature sensitivity could be achieved. The intensity ratios were determined according to Equation (5). The selected filters were approximated with a rectangular bandpass with a center wavelength ± 0.5 FWHM. In general, fuel–dye mixtures are only detectable in the liquid phase. Lower temperatures lead to a frozen fuel–dye mixture and cause significant extinction effects within the test cell (with a path length of 19.1 mm), which drastically weakens the laser beam. The detected emission spectra of E100 and Jet A-1 (183 K) were the lowest, followed by E20 and E40 (193 K) and isooctane (203 K). The detection of the fluorescence signals of frozen liquids could only be realized if the extinction length were to be significantly reduced. This would require another LIF chamber geometry.

The emission spectra of isooctane were characterized by a double peak. A decreased temperature shifted the left flank and the peaks toward higher wavelengths (11.7 nm within [293 K, 203 K]). The spectral shift increased with lower temperatures, and the left peak became less pronounced with a decreasing temperature.

The biofuel blends E20 and E40 showed similar behavior. The double peak of Nile red in isooctane transformed with the ethanol admixture to a single peak. This behavior was already reported in [44] for room-temperature conditions. A decrease in temperature shifted the left flank and the peak (E20: 9.5 nm within [293 K, 223 K]; E40: 6.5 nm within [293 K, 233 K]) toward higher wavelengths. The spectral shift decreased with lower temperatures. Since the emission behavior changed significantly at 213 K (E20) and 223 K (E40), which can also be seen in the sensitivity curves (see Figure 7f), only temperatures up to 223 K (E20) and 233 K (E40) could be considered for further analysis.

The emission spectra of Eosin-Y in E100 were characterized by a single peak, like E20 and E40. A temperature decrease led to a shift in the right flank and the peak toward shorter wavelengths (11.3 nm within [293 K, 183 K]) with decreasing temperature. This was different from the temperature-dependent emission of Nile red in various solvents (see the discussion below). The spectral shift remained constant until 213 K and increased afterward with decreasing temperature. At lower temperatures, a saddle point with a minor maximum was formed (<193 K). This distinct spectral behavior of Eosin-Y was not reported in the literature before.

At moderate temperatures, the emission spectra of Jet A-1 were characterized by a double peak, like isooctane. Lower temperatures led to a decrease in the left peak, and only the right peak remained (<233 K). The two flanks and the peak were shifted toward higher wavelengths with decreasing temperature. The right peak was shifted with decreasing temperature by 47 nm [293 K, 183 K] toward higher wavelengths.

A red-shift and broadening of the fluorescence spectrum with increasing temperature is common for most aromatic species [48] and organic fluorescence dyes [26]. With increased temperatures, the molecules may occupy higher vibrational levels of the excited states. Fluorescence from these excited state levels may lead to a population of highly excited vibrational levels in the ground state. This results in a decreased fluorescence photon energy, explaining the spectral red-shift and broadening of the fluorescence spectrum. This effect was obvious for Eosin-Y in ethanol. However, Nile red showed the opposite trend, which can be explained by the different energy transfer and de-excitation pathways of this molecule after excitation, such as internal conversion (IC) or intersystem crossing (ISC) and phosphorescence. The reason for this blue-shift in the emission spectrum is not accessible via the present measurements as the time scales of the radiative and non-radiative processes of Nile red in various solvents are unknown. Usually, fluorescence decay times under various conditions have to be analyzed for deeper insights into these specific photo-physical effects. However, these measurements are not available in the literature for Nile red and Eosin-Y and will be part of our future work. For Nile red dissolved in isooctane, E20, and E40, the left flanks of the emission spectra were shifted to lower wavelengths with increasing temperature. This can also be explained by the similar shift in the absorption spectra with temperature in that wavelength region [28]. Consequently, the reabsorption in that spectral range was reduced. However, these temperature-dependent absorption measurements were not part of the present study on the fuel–dye mixtures.

Similar emission behavior of the dyes studied has already been reported in one of our previous investigations [50], where the peaks for Eosin-Y (dissolved in water; water + 20 vol.% glycol and water + 40 vol.% glycol) were shifted toward longer wavelengths with increasing temperature as well, while the peaks of Nile red dissolved in oils such as Fragoltherm F12 and Marlotherm LH were shifted toward shorter wavelengths.

**Figure 8:**
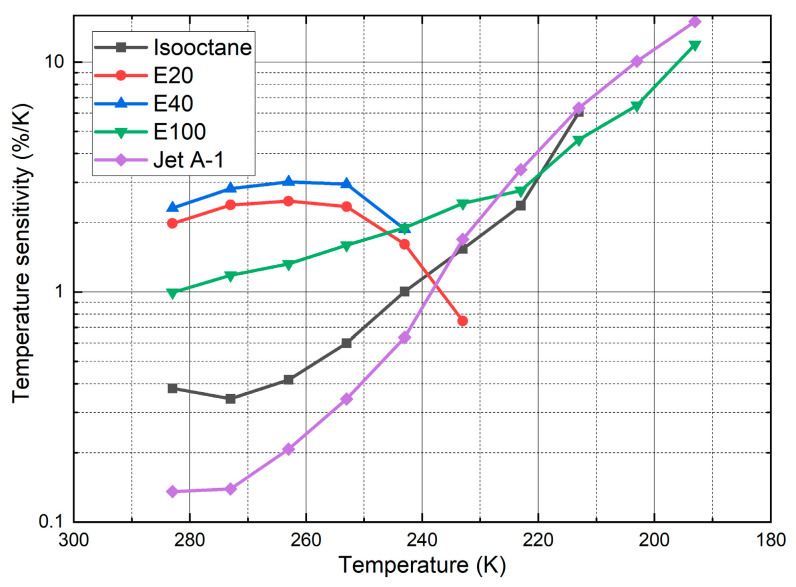
Temperature sensitivity for various fuel–dye mixtures.

The relative temperature sensitivities are shown in Figure 8. The measurements revealed an increase in sensitivity for isooctane, E100, and Jet A-1 at lower temperatures (<270 K), while E40 and E20 showed the opposite behavior.

In summary, the highest average temperature sensitivity (average over the whole temperature interval) was achieved for Jet A-1 (5.2%/K), followed by E100 (4.95%/K), E40 (4.07%/K), E20 (3.23%/K)m and isooctane (3.07%/K). The temperature sensitivities of isooctane (0.38%/K @ 283 K; 6.06%/K @ 213 K), E100 (0.99%/K @ 283 K; 11.97%/K @ 193 K), and Jet A-1 (0.14%/K @ 283 K; 15.03%/K @ 193 K) increased with decreasing temperature. At moderate temperatures (283 K, 243 K), the sensitivities of isooctane and Jet A-1 were below 1%/K and less reliable for temperature determination in that interval. The sensitivities of E20 and E40 showed the opposite behavior. The sensitivities (E20: 1.99%/K @ 283 K, 0.75%/K @ 233 K; E40: 2.32%/K @ 283 K, 1.87%/K @ 243 K) initially slightly increased between 283 K and 263 K with decreasing temperature and rapidly decreased afterward. The high temperature sensitivity levels of E20, E40, and E100 enabled a reliable temperature determination across the whole temperature interval. The current temperature-dependent measurements are in agreement with the earlier investigation by the group at moderate temperatures (263 K–343 K), which also showed larger temperature effects of the LIF signals for kerosene and its biofuel blends (HEFA and farnesane) in comparison with E20 (80% “toliso”, which is a mixture containing toluene and isooctane, and 20% ethanol) and E40 (60% “toliso”) [28]. However, it should be noted that the filter selection was optimized for high temperature sensitivities at very low temperatures. Nile red in isooctane and Jet A exhibited very low temperature sensitivities in the temperature range of 243–293 K.

The derived sensitivities were comparable to those of other dyes in different solvents reported in the literature in this field (however, not at those cryogenic temperatures). The presented two-color LIF concept should ensure a reliable temperature determination of the investigated mixtures (liquid phase). For example, Prenting et al. characterized dyes for two-color LIF thermometry (in the range of 296 K–348 K) for spray applications [27]. They calculated a temperature sensitivity of coumarin in ethanol of 1.2%/K. Palmer et al. measured the temperature (285 K–321 K) of micro-droplets and derived a temperature sensitivity of pyrromethene in ethanol of 1.2%/K [39]. Koegl et al. characterized suitable tracers for two-color thermometry for cooling applications for electric and electronic components. They determined the temperature sensitivities of Eosin-Y in water (1.62%/K), a water–glycol mixture (80 vol.% water, 20 vol.% glycol; 1.80%/K), and Nile red in Marlotherm LH (4.22%/K) and Fragoltherm F12 (1.99%/K) [50]. Vetrano et al. calculated the temperature sensitivity of rhodamine B in ethanol (297 K–328 K) to be 0.7%/K [33].

To achieve the best possible fit, two different fit approaches were utilized. The temperature for isooctane, E20, E100, and Jet A-1 can be determined according to the following exponential equation:

(3) $$T_{\mathrm{Liquid}}={A_{1}}^{\frac{-r_{\mathrm{Temperature}}}{t_{1}}}+{A_{2}}^{\frac{-r_{\mathrm{Temperature}}}{t_{2}}}+{A_{3}}^{\frac{-r_{\mathrm{Temperature}}}{t_{3}}}+y_{0}$$

The temperature for E40 can be derived with the following polynomial equation:

(4) $$T_{\mathrm{Liquid}}(K)=p_{1}\cdot r_{\mathrm{Temperature}}^{4}+p_{2}\cdot r_{\mathrm{Temperature}}^{3}+p_{3}\cdot r_{\mathrm{Temperature}}^{2}+p_{4}\cdot r_{\mathrm{Temperature}}+p_{5}$$

The fit parameters for the investigated fuels and the corresponding coefficients of determination R^2^ are shown in Table 4 and Table 5. The ratio *r*_Temperature_ can be determined according to Equation (5). Herein, *τ* is the transmission curve of the respective filters, and *I*_LIF_ is the detected fluorescence signal (when no bandpass filters are used):

(5) $$r_{\mathrm{Temperature}}=\frac{\sum \tau_{CH1}\cdot I_{LIF}}{\sum \tau_{CH2}\cdot I_{LIF}}$$

**Figure 9:**
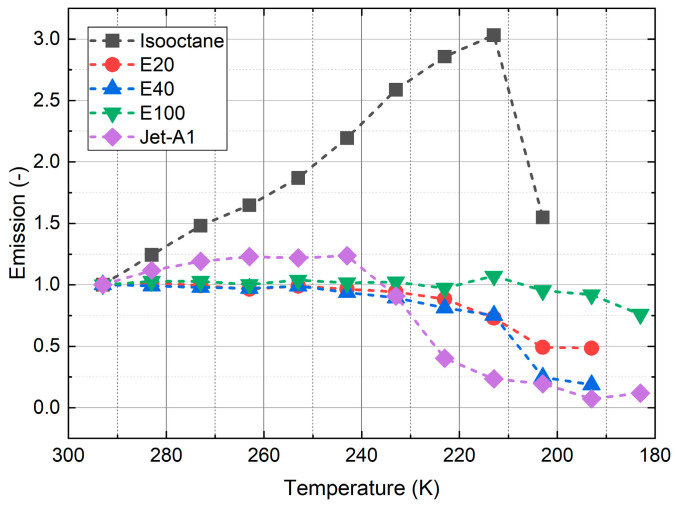
Temperature-dependent integrated signal intensities (536 nm–800 nm) for various fuel–dye mixtures.

The previous section dealt with normalized spectra (see Figure 7) to clarify the effects of temperature on the LIF signals. Furthermore, in the application of the technique, the signal dynamics play an important role. Conventional scientific sCMOS cameras (e.g., Imager, LaVision, Goettingen, Germany) can handle a signal dynamic of 82.7 dB (1:13.640) [62]. The temperature-dependent integrated signals of the individual spectra between 536 nm and 800 nm (shorter wavelengths were not considered to exclude the influence of the excitation laser peak) of the investigated fuel–dye mixtures are presented in Figure 9. Isooctane doped with Nile red showed an increase in the emission signal with decreasing temperature (down to 213 K), while the signals of the other dye–fuel mixtures moderately decreased with lower temperatures. The change in the emission signal was in the range of factor +3 to −14. This dynamic range can be handled with conventional available scientific sCMOS cameras.

A camera-based detection system with two detection channels (equipped with bandpass filters), similar to [21], should enable the temperature determination of fuel films and sprays. The presented measurements and calculations ensure the suitability of the suggested dyes and filters. Since the camera-based detection system has a different efficiency (i.e., quantum efficiency, optical efficiency of the whole setup including the mirror and lenses, etc.), the temperature calibration has to be repeated to ensure adequate accuracy and precision of the future planar thermometry setup under low temperatures and cryogenic conditions.

## 5. Conclusions and Future Work

Absorption and emission spectroscopy of Nile red and Eosin-Y dissolved in various fuels at low and cryogenic temperatures was conducted in order to develop a two-color thermometry technique based on laser-induced fluorescence. The fluorescence dye Nile red was used for isooctane, E20, E40, and Jet A-1, and Eosin-Y was applied as a tracer for ethanol. First, a low-temperature LIF cell was developed based on cooling with liquid nitrogen. With the cell, inner temperatures down to 153 K could be achieved. Afterward, the influence of the tracer concentration on the spectral behavior (linearity of emission/absorption) was studied. Herein, the investigation revealed a linear emission behavior up to a dye concentration of 4.69 mg/L in the present setup. Photo-dissociation effects could be neglected for all investigated fuel–dye mixtures for the realized short measurement times. Temperature-dependent laser-induced fluorescence spectra (down to 183 K for single fuels depending on the freezing point) were recorded. Frozen fuel–dye mixtures cause significant extinction effects, prevent sufficient signal detection, and define the temperature limit.

The temperature-dependent fluorescence measurements revealed different behaviors for the fuels. The left flanks and the peaks of the emission spectra of Nile red dissolved in isooctane and Jet A-1 were shifted with decreasing temperature toward higher wavelengths. The biofuel blends E20 and E40 doped with Nile red showed similar behavior. A temperature decrease for Eosin-Y in E100 led to a shift in the right flank and the peak toward shorter wavelengths with decreasing temperature. The spectral shift remained constant (for temperatures of ≥213 K) and increased with decreasing temperatures (<213 K). At decreasing temperatures, a saddle point with a lower maximum was formed (<193 K).

These shifts in the emission spectra were exploited for thermometry based on two-color LIF, which was based on intensity ratios realized with bandpass filters selected for very low-temperature measurements. The suggested bandpass filters enabled an overall high temperature-sensitive intensity ratio (the average over the temperature interval) with the largest sensitivity for Jet A-1 (5.2%/K), followed by E100 (4.95%/K), E40 (4.07%/K), E20 (3.23%/K), and isooctane (3.07%/K). The high temperature-dependent sensitivity levels of E20, E40, and E100 enable a reliable temperature determination within the whole temperature interval studied. Isooctane and Jet A-1 showed sensitivities below 1%/K at moderate temperatures (283 K, 243 K) and may be less reliable for temperature determination in that interval.

These averaged determined sensitivities are comparable to the literature data on other commonly used LIF tracers. Despite this, those sensitivities were not determined under cryogenic conditions. Consequently, the conducted measurements should enable the reliable temperature determination of fuel films and sprays at extremely low temperatures with adequate illumination and a two-color camera-based detection system. The construction of a low-temperature test chamber and the application of planar diagnostics for automotive and aerospace applications will be part of our future work. Furthermore, the specific photo-physical behavior of Nile red and Eosin-Y must be analyzed in more detail under various conditions for the development and optimization of diagnostic tools. For this purpose, fluorescence decay time measurements under various conditions are necessary (see, e.g., [48]).

## Figures and Tables

**Table 1:** UV/VIS spectrometer specifications and settings.

Light sources	Halogen/deuterium lamps
Wavelength interval	190 nm–900 nm
Bandwidth	2 nm
Scan speed	200 nm/min
Measurement temperature	293 K

**Table 2:** Physical properties of the investigated fuels [52,53,54,55,56,57,58,59,60].

Property	Unit	Isooctane	Ethanol	Jet A-1
H/C ratio/O/C ratio	-	2.25/-	3/0.5	1.92/-
Boiling point or range	K	372	351	478–573
Density at 293 K and 0.1 MPa	g/cm^3^	0.72	0.79	0.79
Dynamic viscosity at 0.1 MPa and 298 K	mPa·s	0.47	1.1	1.33 (@293 K)
Surface tension at 293 K	N/m	0.019	0.022	0.027
Heat of vaporization at 293 K	kJ/kg	297	904	300–375
Stoichiometric air–fuel ratio	kg/kg	15.2	9	~15
Lower heating value	MJ/kg	44.3	26.8	43.45

**Table 3:** Selected bandpass filters (10 nm FWHM) with corresponding article numbers (SNs (stock numbers) and Edmund optics).

	Filter 1		Filter 2	
Solvent	CWL (nm)	SN	CWL (nm)	SN
Isooctane	632	#65-166	560	#88-011
E20	640	#65-168	600	#65-163
E40	640	#65-168	600	#65-163
E100	540	#65-157	568	#65-160
Jet A-1	650	#65-170	589	#65-162

**Table 4:** Fit parameters for the two-color approach of isooctane, E20, E100, and Jet A-1.

Fuel	y_0_	A_1_	t_1_	A_2_	t_2_	A_3_	t_3_	R^2^	Valid
Isooctane	199.73	−7914.23	0.34	13,478.36	0.25	2158.91	0.46	0.9975	(203 K–293 K)
E20	458.26	−72.23	−4.48	0.00	−0.06	−62.26	−4.49	0.9962	(223 K–293 K)
E100	176.24	160.98	0.63	69.06	0.63	118.03	1.89	0.9996	(183 K–293 K)
Jet A-1	177.18	3.26 × 10^7^	0.07	2.75 × 10^9^	0.05	88.84	2.17	0.9991	(183 K–293 K)

**Table 5:** Fit parameters for the two-color approach of E40.

Fuel	p_1_	p_2_	p_3_	p_4_	p_5_	R^2^	Valid
E40	−27.40	164.83	−350.82	273.10	232.99	0.9974	(233 K–293 K)

## Data Availability

Dataset available on request from the authors.
